# A comparison of earlier and enhanced rehabilitation of mechanically ventilated patients in critical care compared to standard care (REHAB): study protocol for a single-site randomised controlled feasibility trial

**DOI:** 10.1186/s40814-017-0131-1

**Published:** 2017-04-17

**Authors:** Catherine Snelson, Charlotte Jones, Gemma Atkins, James Hodson, Tony Whitehouse, Tonny Veenith, David Thickett, Emma Reeves, Aisling McLaughlin, Lauren Cooper, David McWilliams

**Affiliations:** 1 Department of Critical Care, University Hospitals Birmingham NHS Foundation Trust, Edgbaston, Birmingham, UK; 2 Therapy Services, University Hospitals Birmingham NHS Foundation Trust, Edgbaston, Birmingham, UK; 3 Department of Statistics, University Hospitals Birmingham NHS Foundation Trust, Edgbaston, Birmingham, UK; 4 Institute of Inflammation and Ageing, College of Medical and Dental Sciences, Centre for Translational Inflammation Research, University of Birmingham Laboratories, Queen Elizabeth Hospital Birmingham, Birmingham, UK; 5 National Institute for Health Research Surgical Reconstruction and Microbiology Research Centre, Queen Elizabeth Hospital Birmingham, Birmingham, UK

**Keywords:** Critical care, Mechanical ventilation, Rehabilitation, Physiotherapy

## Abstract

**Background:**

Mortality from critical illness is improving, but survivors suffer from prolonged weakness and psychological and cognitive impairments. Maximising the recovery after critical illness has been highlighted as a research priority, especially in relation to an ageing population who present with higher rates of pre-morbid disability. Small studies have shown that starting rehabilitation early within the intensive care unit (ICU) improves short-term outcomes. Systematic reviews have highlighted the need for robust multicentre randomised controlled trials with longer term follow-up.

**Methods:**

The study design is a randomised controlled study to explore the feasibility of providing earlier and enhanced rehabilitation to mechanically ventilated patients at high risk of ICU-acquired weakness within the ICU. The rehabilitation intervention involves a structured programme, with progression along a functionally based mobility protocol according to set safety criteria. The overall aim of the intervention is to commence mobilisation at an earlier time point in the patient’s illness and increase mobility of the patient through their recovery trajectory. Participants will be randomised to enhanced rehabilitation or standard care, with the aim of recruiting at least 100 patients over 16 months. The trial design will assess recruitment and consent rates from eligible patients, compliance with the intervention, and assess a range of possible outcome measures for use in a definitive trial, with follow-up continuing for 12 months post hospital discharge.

**Discussion:**

This study will evaluate the feasibility of providing an earlier and enhanced rehabilitation intervention to mechanically ventilated patients in critical care. We will identify strengths and weaknesses of the proposed protocol and the utility and characteristics of the outcome measures. The results from this study will inform the design of a phase III multicentre trial of enhanced rehabilitation for critically ill adults.

**Trial registration:**

ISRCTN90103222, 13/08/2015; retrospectively registered.

## Background

Survivors of critical illness suffer from high mortality and significant physical, cognitive and psychological morbidity following discharge from the intensive care unit (ICU), a process now termed “postintensive care syndrome” [1]. These effects can last for many years after hospital discharge [2], with a significant socioeconomic burden upon patients and their caregivers [3]. In one study, among survivors of severe sepsis who lived independently prior to their illness and survived hospitalisation, approximately one third had died at 6 months and another third had problems with mobility or self-care and were unable to live independently [4]. Older ICU survivors in particular suffer prolonged and persistent decline in cognitive and physical function [5] with those with a length of stay more than 2 weeks at highest risk for 1-year mortality and disability [6]. Older age is an independent predictor of mortality following critical illness even after controlling for severity of critical illness and co-morbidities, although the burden of pre-morbid disease and disability is a factor [7, 8].

Muscle weakness experienced by ICU patients is multifactorial; sarcopenia may exist from pre-morbid conditions. There is acquired disuse atrophy from bed rest [9] and ICU-acquired weakness (ICUAW) may develop [10]. ICUAW is a diffuse, symmetric and generalized muscle weakness that develops after the onset of critical illness without another identifiable cause and is a combination of critical illness polyneuropathy and critical illness myopathy based on electromyography and nerve conduction studies [11]. Muscle wasting occurs early and rapidly during the first week of critical illness, correlates with the degree of organ failure [12] and is associated with failure to wean from ventilation and increased in-hospital mortality [13, 14]. Preventing the physical consequences of critical illness and supporting recovery from intensive care has therefore been identified as a high priority area for critical care research [15].

In small studies, early and enhanced rehabilitation on the ICU has been demonstrated to have beneficial effects on muscle strength, physical function, health-related quality of life, ventilator-free days and length of stay in ICU and hospital. The effects on longer term outcomes are still uncertain [16, 17]. A Cochrane review of ICUAW has suggested that further large randomised controlled trials are needed to explore the role of early rehabilitation in prevention and treatment [18]. Surveys of practice have demonstrated significant international variation in the delivery of rehabilitation with presence of a dedicated physiotherapist, daily goal setting and multidisciplinary ward rounds being associated with early mobility practices [19, 20]. Recently published randomised controlled trials of early rehabilitation on the ICU have failed to show long-term significant benefits, but they have been limited by recruiting patients with short lengths of stay in the ICU and therefore lower levels of ICUAW or mismatches in the baseline characteristics [21–23].

Indeed, the term “early” has yet to be defined with onset of interventions varying by as much as 1 week [24]. The important factor may be “earlier” interventions, whereby mobilisation can be initiated at a more acute stage of the patient’s illness than would otherwise occur. After 10 days in the ICU, the admission diagnosis and physiological derangement become less important than simple antecedent patient characteristics such as age, sex and chronic health status in determining outcome and although only representing 5% of all ICU admissions, these patients with “persistent critical illness” consume significant resource and require dedicated future research [25].

Our group has recently published the results of a quality improvement project, where a new supportive rehabilitation team was created with a focus on promoting earlier and enhanced rehabilitation for patients at high risk of prolonged ICU and hospital stays [26]. The introduction of the team led to a significant improvement in mobility at ICU discharge, and this was associated with a significant reduction in ICU length of stay (geometric mean of 16.9 versus 14.4 days, *p* = 0.007), ventilator days (geometric mean of 11.7 versus 9.3 days, *p* = 0.05) and in-hospital mortality (39 versus 28%, *p* = 0.028). However, only a small proportion of the eligible ICU patients were treated by the team and unmeasured confounding factors may be impacting on these results. As such, it is unclear whether the results would be transferable to other centres.

### Aims and objectives

The aim of this feasibility study is to inform the design of a future multicentre phase III randomised controlled trial of enhanced rehabilitation compared to standard care in patients mechanically ventilated for 5 days or more. This time point has been specifically chosen to target a patient cohort at greatest risk for ICUAW and persistent critical illness [25, 27]. Specifically, the objectives are to:

1. Estimate rates of recruitment and consent from eligible patients and to describe the baseline characteristics of the participants in terms of co-morbidities, physical function and illness severity.
2. Test the rehabilitation intervention in terms of compliance, differentiation from standard care and ability to increase mobility levels at ICU discharge.
3. Estimate retention of participants and response rates to follow-up questionnaires.
4. Evaluate a range of clinical and patient-reported outcome measures to aid selection of the most appropriate primary outcome measure for a definitive trial, with estimates of variance for sample size calculation.

## Methods

### Design

**Fig. 1:**
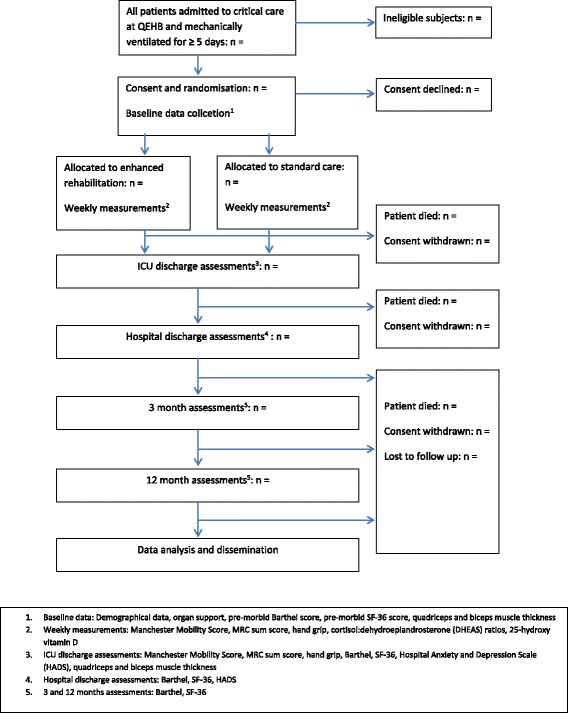
Trial flowchart for REHAB

REHAB is a randomised, controlled, single-site feasibility study comparing enhanced rehabilitation to standard care in patients mechanically ventilated for 5 days or more. A study overview is shown in Fig. 1.

### Participants

Eligible patients will be those who are admitted to the critical care unit at Queen Elizabeth Hospital Birmingham (QEHB) and have been mechanically ventilated for ≥5 days between the 1st June 2015 and 30th September 2016. Patients will be excluded if they have an orthopaedic contraindication to mobilisation (e.g. pelvic or spinal fractures), have an already established rehabilitation pathways (e.g. amputees), have a poor level of mobility prior to ICU admission (defined as <10 yards unassisted), have a pre-existing neuromuscular disease, have a profound acquired neurological deficit (defined as not expected to regain a Glasgow Coma Score of at least 13), have received mechanical ventilation for >48 h at another facility prior to admission to QEHB, have a total hospital length of stay of >7 days prior to the onset of mechanical ventilation or have been previously recruited into the study. Patients will also be excluded if withdrawal of treatment is expected within the next 24 h.

### Recruitment and consent

Potential participants will be identified on daily ward rounds by the critical care research nursing team. Recruitment will take place on day 4 of mechanical ventilation provided no plan is in place for extubation in the following 24 h, with a cutoff for consent and randomisation of a maximum of day 6 of mechanical ventilation. This is to allow rehabilitation to commence as early as possible. Written informed consent will be gained, but the majority of patients will have altered consciousness caused by illness and therapeutic sedation and will lack capacity. We will therefore approach a personal consultee in a face-to-face meeting, who may be a relative, partner or close friend, or an independent registered medical practitioner if no personal consultee is available. The principal investigator or research team member with delegated authority will provide the consultee/medical practitioner with information about the study and ask about their opinion of the wishes and feelings of the patient if they had capacity.

All participants will be reviewed regularly to determine recovery and capacity. Consent to continue in the study will be sought as soon as the patient regains capacity and it is deemed appropriate to discuss the study. This will usually take place just prior to or after intensive care discharge. If the patient declines ongoing participation in the study, the patient will receive any further rehabilitation as decided by the responsible clinical team. If the patient dies before regaining capacity, or if mental capacity is not regained, the consent provided by the consultee/medical practitioner will stand.

These are the standard consent procedures for clinical trials in an ICU population within the UK and in line with procedures for obtaining consent already in place for other ethically approved, locally conducted ICU trials.

### Ethics/governance

The study received a favourable ethical opinion from the Research Ethics Committee East Midlands—Nottingham 1 (reference15/EM/0114) on the 8th April 2015. Approval to extend recruitment from 12 to 16 months was obtained on the 18th March 2016. Sponsorship will be provided by the University Hospitals Birmingham NHS Foundation Trust, and annual reporting of trial progress will provided to the sponsor and the Research Ethics Committee. Trial oversight and review of serious adverse events will be provided by the Critical Care Research Management Group which meets monthly.

### Randomisation and blinding

Once authorisation has been obtained for the patient to participate in the study, the patient will be allocated a unique participant study number and baseline demographical data, details of current levels of organ support and Sequential Organ Failure Assessment (SOFA) score will be recorded. The electronic patient record will be flagged to record recruitment. Patients will be assigned to enhanced rehabilitation or standard care using a computer-based stratified blocked randomisation. Stratification is into four groups, based on the combinations of age (<50 versus ≥50 years) and SOFA score (<9 versus ≥9). Within each group, a pre-specified block size will be used, in order to prevent runs of consecutive patients being assigned to the same group, to minimise the risk of introducing bias. The stratification groups have been chosen due to the effect of the severity of organ failure and age on physical outcomes [7, 12].

Given the nature of the intervention, it is not possible to blind physiotherapists or participants to group allocation. Recruitment, randomisation and completion of assessments will be undertaken by the research nursing team, who are independent from the therapy team who will be delivering the rehabilitation. Complete blinding of the nursing team cannot be assured within a single-site study, and evaluation of the chosen outcome measure will require a blinded assessor in a future large-scale trial.

### Study interventions

#### Standard care

Regardless of the day of admission, all patients are assessed by the physiotherapy team within 24 h of admission to critical care. Baseline assessments include reason for admission and relevant pre-existing conditions. Patients continue to be seen on a daily basis on weekdays, with rehabilitation commencing based on the individual physiotherapists own clinical reasoning. Physiotherapy provision is funded at a ratio of 1 physiotherapist to 10 patients, with an average treatment time of 30–45 min per patient per day Monday to Friday with one physiotherapist. Rehabilitation provision is individually prioritised with no set structure or format for rehabilitation in place, and only limited rehabilitation currently takes place whilst the patient is receiving mechanical ventilation. When the patient is discharged from critical care, a verbal handover is provided to the receiving therapist. Rehabilitation is then continued until the patient is deemed safe for discharge from the acute care hospital. No further input is provided by the critical care physiotherapy team.

#### Enhanced rehabilitation (intervention group)

The aim of the intervention is to increase the amount, quality and structure of physical rehabilitation received by patients within the ICU and to provide this at an earlier stage of the patient’s acute illness as measured against daily organ failure assessment scores. Subjects assigned to the intervention group will have all physiotherapy sessions delivered by members of the critical care rehabilitation team in order to minimise contamination between groups.

Following recruitment and randomisation, each subject will have a physiotherapy key worker assigned who will complete a standardised comprehensive assessment. This will provide background information regarding physical function, any psychological history and pre-admission exercise capacity to allow an individually tailored rehabilitation programme to be devised. The rehabilitation plan will be documented and displayed in the subject’s bed space to aid communication and track daily achievements. Subjects will also be discussed at weekly goal-setting meetings to review progress and update treatment plans. To facilitate ongoing rehabilitation following critical care discharge, both verbal and written handovers will be provided to ward therapy staff. For patients achieving a Manchester Mobility Score (MMS) [28] of ≤4 at critical care discharge, ongoing rehabilitation will continue to be provided by the key worker in conjunction with the ward therapists for the first week following discharge from critical care. This will aim to ensure a seamless handover of care and maximise ongoing rehabilitation.

**Fig. 2:**
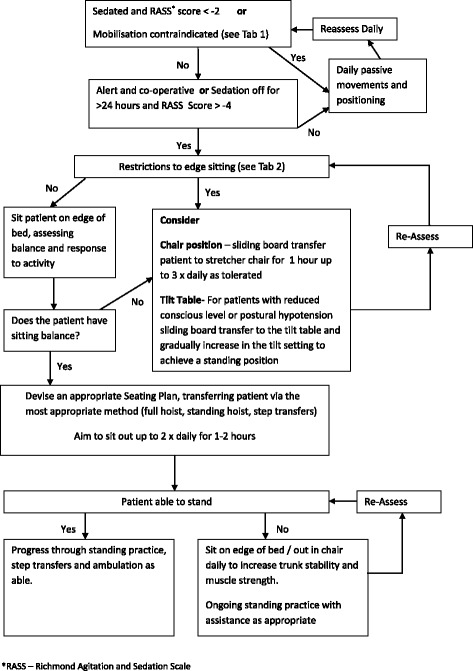
Flow chart for earlier and enhanced rehabilitation

The process of structured critical care rehabilitation which will be adopted for the management of the intervention group is shown in Fig. 2 with specific safety criteria in Tables 1 and 2. To summarise, during the acute phase of a patient’s illness whilst they are still sedated and/or paralysed, rehabilitation is confined to the bed with daily passive movements and positioning. As soon as patients are stable and awake enough to commence more active mobilisation, they are assessed by sitting on the edge of the bed, allowing an evaluation to be made of sitting balance, exercise capacity and physiological stability. This may be performed with endotracheal tubes or tracheostomies still in situ, and whilst the patient is still on ventilatory and/or renal support and low levels of vasopressor or inotropic support. Following this assessment, a rehabilitation plan is formulated which includes the patient sitting out of bed in a chair using the most appropriate method for transfers. More active rehabilitation is administered as the patient improves to progress to standing, transferring and walking.

**Table 1:** Contraindications to mobilise

• Significant dose of vasoactive agents (e.g. >0.2 mcg/kg/min noradrenaline or equivalent)	
• Mechanically ventilated with FiO_2_ >0.6 and/or PEEP >12 cmH_2_O or acutely worsening respiratory failure	
• Acute neurological event	
• Unstable spine or extremity fractures with contraindications to mobilise	
• Active bleeding process

**Table 2:** Restrictions to edge sitting

• Small dose of vasoactive agents (e.g. 0.1–0.2 mcg/kg/min noradrenaline or equivalent) required for haemodynamic stability	
• Mechanically ventilated with FiO_2_ >0.6 and/or PEEP >10 cmH_2_O	
• Poor tolerance of endotracheal tube	
• Open abdomen or high risk for dehiscence—liaise with surgical team prior to mobilising	
• Haemofiltration via a femoral line	

#### Data collection

At recruitment, demographic data including age, sex, reason for ICU admission, illness severity score (APACHE II) [29] and the Charlson co-morbidity score [30] will be recorded, and retrospective estimates of the Barthel [31] and Short Form-36 scores [32] will be taken from family members. Daily data will be collected until discharge from the ICU on SOFA score, sedation use, delirium and nutritional intake.

All medical care will be at the discretion of the responsible intensivist. Rehabilitation interventions by all members of the clinical team at each stage of the patient pathway will be carefully recorded, with analysis of the reasons for missed rehabilitation sessions performed. In accordance with current unit practice, all patients with a length of stay over 14 days will be discussed at weekly multidisciplinary team meetings which include consultant medical staff, senior nursing staff, speech and language therapists and occupational therapists, with collaborative treatment goals set, reviewed and updated.

Participants will have muscle thickness of quadriceps and biceps measured on recruitment and ICU discharge using ultrasound. Blood will be drawn between 8 am and 10 am on recruitment and weekly until hospital discharge or 6 weeks (whichever is the sooner) for 25-hydroxy vitamin D (all patients) and for cortisol, dehydroepiandrosterone-sulphate (DHEAS) and cortisol metabolites (all patients not receiving steroids as part of their medical management). A subset of patients will also have blood taken at recruitment, week 1 and week 2 for cytokine and neutrophil function. This is to provide exploratory data about the potential impact of rehabilitation on muscle thickness and immune-endocrine function in chronic critical illness.

### Outcome measures

This study will evaluate the feasibility of the enhanced rehabilitation intervention in terms of recruitment process, compliance and differentiation from standard care and provide pilot data as to outcome measures and variance for a phase III trial. For the recruitment process, this will be the proportion of eligible patients who are recruited and then complete all study assessments. Within a single centre, there is a significant risk of changing practice within the control group over the course of the study. Compliance and differentiation of groups will be assessed by process measures such as the daily maximum MMS achieved compared to the level of organ support required, time to first mobilisation (defined as sitting on the edge of the bed (MMS ≥ 2)), proportion of patients who have weekly goals set and documented treatment plans, dose of physiotherapy in terms of therapy time, recording of reasons for missed sessions and mobility level at ICU discharge.

Outcome measures to be evaluated for potential use within a definitive trial are as follows:

- ICU and hospital length of stay and mortality, as enhanced rehabilitation has been shown to be associated with reduced length of stays and mortality in published literature [16, 17].
- Medical Research Council (MRC) sum score and hand grip strength as a measure of ICU-acquired weakness [33].
- Functional status as assessed by the Barthel index score at ICU and hospital discharge, 3 and 12 months [31].
- Health-related quality of life as assessed by SF-36 at ICU and hospital discharge, 3 and 12 months [32, 34].
- Anxiety and depression as assessed by the Hospital Anxiety and Depression Scale score at ICU and hospital discharge [35, 36].

Completion of the Barthel, SF-36 and HADS scores will be co-ordinated by the research nursing team. Three- and twelve-month assessments will be via telephone interview, with a follow-up sent by post if no response is received. NHS records will be accessed by the nursing team to ensure that no deceased patients are contacted.

To maximise the scientific value of the study, muscle thickness of quadriceps and biceps will be measured by ultrasound at recruitment and ICU discharge to explore whether enhanced rehabilitation may attenuate the loss of muscle mass known to occur in critical illness [12]. Blood samples for the analysis of 25-hydroxy vitamin D, cortisol, DHEAS, cytokine and neutrophil function will be taken to provide hypothesis generating pilot data as to how rehabilitation may affect immunoendocrine function.

A summary of the time schedule of enrolment and assessments is summarised in Table 3.

**Table 3:** Time schedule of enrolment and assessments for participants

	Enrolment	Allocation to intervention	ICU stay	ICU discharge	Ward stay	Hospital discharge	Follow-up 3 months	Follow-up 12 months
Eligibility screen	x							
Informed consent	x			x				
Demographic and APACHE II score		x						
Charlson co-morbidity score		x						
SOFA score		x	Daily					
Sedation use			Daily					
CAM-ICU delirium assessment			Daily					
Dose of physiotherapy			Daily		Daily			
Nutritional intake			Daily					
MMS		x	Daily	x				
Barthel		x		x		x	x	x
SF-36		x		x		x	x	x
HADS score				x		x		
MRC sum score			Weekly		Weekly			
Muscle USS		x		x				
Grip strength			Weekly		Weekly			
Blood tests		x	Weekly		Weekly			
Urine sample		x	Week 1					

### Data management

All data for an individual participant will be collected by the principal investigator or their delegated nominees and recorded in the case report form (CRF). Participant identification in the CRF will be through their unique participant study number allocated at the time of randomization and initials. Data from the CRF will be entered onto a secure password protected database held on a trust computer. Due care will be taken to ensure data safety and integrity and compliance with the UK Data Protection Act 1998. Study documentation and data will be archived for at least 6 years after completion.

### Sample size

As this is a feasibility study, no sample size calculation has been performed. Over 300 patients per year are mechanically ventilated for more than 5 days at QEHB. After taking account of patients who have one or more of the exclusion criteria, we expect to recruit over 100 patients during the 16 months of the study. As we are recruiting critically ill patients, we expect a high level of in-hospital mortality of up to 30%.

### Statistical analysis

The main analytical aims of the study are to measure the demographics of the cohort and to estimate the recruitment, compliance and retention rates, to use as a basis of the design and sample size determination of future studies. Analysis of the screening log will provide data regarding the number of potential eligible patients recruited and the reasons for non-recruitment. The number of withdrawals and reasons for withdrawals will be assessed. Patient demographics will be summarised using rates, means and standard deviations or medians and ranges/interquartile ranges, as applicable.

The data collected will also be used to perform a preliminary analysis, to identify whether any of the clinical or patient-reported endpoints differ between the two treatment groups. Baseline and demographic factors will initially be compared between the two groups, in order to ensure balance. Withdrawal rates will also be compared, in order to ensure that those patients in the enhanced rehabilitation group are not at a higher risk of leaving the study. Outcome measures will then be described for each group separately to assess whether change over time can be observed in order to identify potential target outcome measures to utilise in future studies. Effect sizes will be reported as estimates with 95% confidence intervals and without *p* values, as the trial is not powered for testing hypotheses about effectiveness.

### Adverse event management

An adverse event is defined as any untoward medical occurrence in a subject and which does not necessarily have a causal relationship with this treatment. A serious adverse event (SAE) is an adverse event that fulfils one or more of the following criteria:

- Results in death
- Is immediately life-threatening
- Requires prolongation of existing hospitalisation
- Results in persistent or significant disability or incapacity
- Is an important medical condition

Serious adverse events will be reviewed by the study team, and likely causality will be assessed and recorded on the SAE form. All SAEs will be notified to the sponsor’s research and development department via the SAE form in the CRF. Only those events classified as probable or definitely related will be reported to the Research Ethics Committee. Because this study is recruiting a population that is already in a life-threatening situation, it is expected that there will be a high rate of SAEs.

## Discussion

Optimising the recovery of survivors of critical illness is an important area of research that has been highlighted as a priority by the intensive care community. Early and enhanced rehabilitation on the ICU has been shown to be safe [37] and to be associated with short-term improvements in clinical outcomes in some studies. Our findings will determine whether recruitment to a full-scale trial of enhanced rehabilitation of mechanically ventilated patients is feasible. They will allow detailed information on the delivery and dose of the intervention to be collected, allowing refinement if necessary. Success of this feasibility study will be judged by whether it is possible to show that a differentiation in dose of therapy between groups is achievable and measurable. Whilst this study is not powered to detect clinically important differences in patient outcomes, it will provide important information about the trajectory of recovery from critical illness and enable sample size calculations to be performed for the design of a phase III trial. A dissemination plan which includes conference presentations and publication in open access peer-reviewed journals following the extended CONSORT principles [38] is in place.

## Trial status

The trial is open and recruiting.

## Abbreviations

CRF: Case report form
DHEAS: Dehydroepiandrosterone-sulphate
ICU: Intensive care unit
MMS: Manchester Mobility Score
QEHB: Queen Elizabeth Hospital Birmingham
SOFA: Sequential Organ Failure Assessment score
